# LRP1 Modulates APP Intraneuronal Transport and Processing in Its Monomeric and Dimeric State

**DOI:** 10.3389/fnmol.2017.00118

**Published:** 2017-04-27

**Authors:** Uta-Mareike Herr, Paul Strecker, Steffen E. Storck, Carolin Thomas, Verena Rabiej, Anne Junker, Sandra Schilling, Nadine Schmidt, C. Marie Dowds, Simone Eggert, Claus U. Pietrzik, Stefan Kins

**Affiliations:** ^1^Institute of Pathobiochemistry, Molecular Neurodegeneration, University Medical Center of the Johannes Gutenberg-University MainzMainz, Germany; ^2^Division of Human Biology and Human Genetics, Technical University of KaiserslauternKaiserslautern, Germany

**Keywords:** amyloid precursor protein (APP), dimerization, transport, low density lipoprotein receptor-related protein 1 (LRP1), processing

## Abstract

The low-density lipoprotein receptor-related protein 1, LRP1, interacts with APP and affects its processing. This is assumed to be mostly caused by the impact of LRP1 on APP endocytosis. More recently, also an interaction of APP and LRP1 early in the secretory pathway was reported whereat retention of LRP1 in the ER leads to decreased APP cell surface levels and in turn, to reduced Aβ secretion. Here, we extended the biochemical and immunocytochemical analyses by showing via live cell imaging analyses in primary neurons that LRP1 and APP are transported only partly in common (one third) but to a higher degree in distinct fast axonal transport vesicles. Interestingly, co-expression of LRP1 and APP caused a change of APP transport velocities, indicating that LRP1 recruits APP to a specific type of fast axonal transport vesicles. In contrast lowered levels of LRP1 facilitated APP transport. We further show that monomeric and dimeric APP exhibit similar transport characteristics and that both are affected by LRP1 in a similar way, by slowing down APP anterograde transport and increasing its endocytosis rate. In line with this, a knockout of LRP1 in CHO cells and in primary neurons caused an increase of monomeric and dimeric APP surface localization and in turn accelerated shedding by meprin β and ADAM10. Notably, a choroid plexus specific LRP1 knockout caused a much higher secretion of sAPP dimers into the cerebrospinal fluid compared to sAPP monomers. Together, our data show that LRP1 functions as a sorting receptor for APP, regulating its cell surface localization and thereby its processing by ADAM10 and meprin β, with the latter exhibiting a preference for APP in its dimeric state.

## Introduction

The amyloid precursor protein (APP) is a type I transmembrane protein that has first been identified related in association with Alzheimer's disease (AD) as representing the precursor of amyloid β (Aβ) peptides (Kang et al., 1987). Those peptides generated by sequential cleavage of APP by β- and γ-secretases were shown to be a major component of senile plaques found in the brains of AD patients (Merz et al., 1983; Masters et al., 1985). Besides its role in AD pathogenesis, APP has been implicated in physiological functions including intracellular signaling, trophic activity in neurons and synapses as well as in synaptic and cell adhesion processes (Baumkötter et al., 2012; Müller and Zheng, 2012). Recent studies revealed that APP can dimerize or oligomerize in *cis*- as well as in *trans*-orientation (Scheuermann et al., 2001; Soba et al., 2005; Munter et al., 2007; Kaden et al., 2009; Wang et al., 2009; Isbert et al., 2012; Baumkötter et al., 2014; Klevanski et al., 2014; Stahl et al., 2014). Remarkably, APP dimers were detected in mouse brains (Soba et al., 2005; Schmidt et al., 2012), indicating that dimer formation occurs *in vivo* under physiological conditions. *Trans*-cellular APP dimerization is assumed to modulate synapse organization (Soba et al., 2005; Wang, 2005; Wang et al., 2009; Isbert et al., 2012; Baumkötter et al., 2014; Klevanski et al., 2014; Stahl et al., 2014). In contrast, APP *cis*-dimerization, that has been shown to occur as early as in the endoplasmic reticulum (ER) (Isbert et al., 2012), has been implicated in processing of APP by α-, β-, and γ-secretases (Munter et al., 2007, 2010; Kaden et al., 2008; Eggert et al., 2009; Libeu et al., 2012; Schmidt et al., 2012; So et al., 2012; Jung et al., 2014). Recently, it has been claimed that efficient processing of APP by α- and β-secretases may depend on its oligomerization state that results in cooperative effects for these allosteric enzymes, influenced by SorLA and possibly also LRP1 (Schmidt et al., 2012). However, whether sAPP dimers are generated *in vivo* in neurons, which secretases are required and what might be the role of LRP1 in this context, is unknown yet.

LRP1, a member of the low density lipoprotein receptor (LDLR) family (Krieger and Herz, 1994), was shown to interact with APP via the N- and C-terminal domain and to affect its processing (Ulery et al., 2000; Pietrzik et al., 2002, 2004). This effect is presumably based on the impact of LRP1 on APP endocytosis (Knauer et al., 1996; Ulery et al., 2000; Pietrzik et al., 2002; Cam et al., 2005). In addition, APP can interact with LRP1 before it is cleaved by furin in the TGN, implying an interaction of APP with LRP1 early in the secretory pathway (Pietrzik et al., 2004). This hypothesis was confirmed in 2008 (Waldron et al., 2008), by using a truncated LRP1-construct (LRP-CT) (Pietrzik et al., 2002) containing a dilysine ER-retention motif (KKAA) capable of binding to APP. The retention of LRP1 in the ER leads to a decrease in Aβ secretion as well as to a decrease in full length APP and CTF levels at the plasma membrane (Waldron et al., 2008).

Here, we extend the analysis of APP transport characteristics and show that LRP1 plays a crucial role in trafficking and processing of monomeric as well as dimeric APP.

## Materials and methods

### Cell culture

Human Embryonic Kidney cells (HEK 293T) were cultured in Dulbecco's Modified Eagle's Medium (DMEM; Thermo Fisher Scientific) supplemented with 10% fetal calf serum (FCS), 1 mM sodium pyruvate (Sigma-Aldrich), 100 units/ml penicillin and 0.1 mg/ml streptomycin (Thermo Fisher Scientific).

Chinese Hamster Ovary cells, either CHO K1 or LRP-deficient CHO 13-5-1 (FitzGerald et al., 1995), were grown in Alpha Minimum Essential Medium (α-MEM; Lonza) supplemented equally.

Primary neurons were extracted from cortices of C57BL/6J or 5xFAD/*Lrp1*^*flox*/*flox*^ mouse embryos at embryonic day 14 as described previously (Maier et al., 2013). Cells were seeded on poly-L-ornithine (100 μg/ml; Sigma-Aldrich) coated 6-well plates or 6 cm dishes, respectively, in a density of 600,000 cells per well or 1,000,000 cells per dish. They were cultured in Neurobasal Medium (Thermo Fisher Scientific) complemented with 100 units/ml penicillin and 0.1 mg/ml streptomycin, 1 x B27 supplement and 1 x GlutaMAX (all Thermo Fisher Scientific).

Primary cortical neurons (PCN) were prepared using E14 embryos from C57BL/6J mice (Janvier) or 5xFAD/*Lrp1*^*flox*/*flox*^ mice as described before (Stahl et al., 2014; Hermey et al., 2015). PCN dissolved in DB1 medium [DMEM with 10% FBS, 0.79% D-glucose and 1 x GlutaMAX (Thermo Fisher Scientific)] were plated on poly-L-lysine (Sigma-Aldrich) coated fluorodishes in a density of 6^*^10^5^/cm^2^. Six hour post plating DB1 was changed and PCN were cultivated in neurobasal medium supplemented with B27 and GlutaMAX (Thermo Fisher Scientific).

Primary hippocampal neurons (PHN), used for APP/LRP live cell imaging, were prepared from P0 pups of C57BL/6J mice and treated in the same way as described for PCN.

All cell types were cultivated at 37°C in an incubator maintaining a relative humidity of over 80% and a CO_2_ level of 5%.

### DNA constructs and cloning

For analyzing the properties of APP *cis*-dimers a human APP695 construct with a dimer-bearing amino acid exchange from lysine (K) to cysteine (C) at position 587 (APP695 K587C) was generated for transient and stable transfections. The plasmid consisting of the human APP695 CDS with the triplet mutation (AAG to TGT) at position 1,761 as well as a C-terminal myc-tag in the vector pLBCX was developed by an overlap extension PCR as described by Isbert et al. (2012). The restriction sites for HindIII and ClaI, which are flanking the myc-tagged, mutated APP sequence, enabled the subcloning of this DNA fragment into the vector pLHCX resulting in the pLHCX-APP695 K587C construct. Hence this construct has the same vector backbone as the also used pLHCX-APP695 wt plasmid (Jäger et al., 2009). APP dimer constructs exhibiting a mutation in the APP internalization motif “YENPTY” (Lai et al., 1995; MarquezSterling et al., 1997) were generated performing a standard PCR followed by restriction and ligation into the pLHCX vector backbone. The plasmid pLHCX-APP695 K587C served as template for PCR using the forward primer 5′-CCCAAGCTTATGCTGCCCGGTTTG-3′, which contains a 5′ HindIII restriction site and the reverse primer 5′-CCATCGATGGTTACAGATCCTCTTCTGAGATGAGTTTTTGTTCGTTCTGCATCTGCTCAAAGAACTTTTCGTAGCCGTTTTCGTAG-3′ exhibiting the mutation in the internalization motif, the myc-epitope and a 3′ ClaI restriction site. The described mutation results in an amino acid exchange from NPTY to NGYE at the C-terminus of the expressed APP695 K587C protein. The amplified DNA fragment was subcloned in frame into the pLHCX vector backbone via the HindIII and ClaI restriction sites. Sequencing of the generated construct authenticated its accuracy. To study the processing of monomeric and dimeric APP by meprin β, HEK 293T cells were co-transfected with either APP695 wt or APP695 K587C and the meprin β HA construct in pLBCX (Schönherr et al., 2016).

For generation of the expression construct encoding the LRP1-GFP fusion protein, the EGFP cDNA was amplified from pcDNA3.1 APP-GFP (Szodorai et al., 2009) using the oligos 5′-TGAGCAGATGCAGAACGTCG-3′ and 5′-GCACAGTCGAGGCTGATCAGC-3′. The PCR product was cloned via flanking BamHI/NotI sites in frame into pLBCX myc-LRP1 and the resulting construct, pLBCX-myc-LRP1-GFP, was validated by sequencing.

### Infections and transfections

The infection of primary cortical neurons (PCN) with an adenoviral vector encoding human APP695 (Yuan et al., 1999) was performed at DIV 7. Cells were incubated with 100 plaque-forming units per cell for 6 h. In contrast, for live cell imaging, PCN or PHN were transiently transfected at DIV 6 using calcium phosphate transfection. A neurobasal medium containing 2% B27 (transfection medium) was prepared and incubated for at least 30 min at 37°C and 5% CO_2_. Meanwhile, the following transfection mix was pipetted (sufficient for two fluorodishes): Solution A containing 75 μl H_2_O dd, 9.5 μl 2.5 M CaCl_2_ and 20 μg DNA; Solution B containing 75 μl 2 × HBS pH 7.07 (274 mM NaCl, 10 mM KCl, 1.4 mM Na_2_HPO_4_, 15 mM D-Glucose, 42 mM HEPES pH 7.1). Solution A was added to Solution B, immediately vortexed for 10 s at maximum speed and incubated for 20 min at RT. Meanwhile, the medium of the cultured neurons was replaced by 2 ml of the previously prepared transfection medium. The old medium was collected for later usage. Afterwards, 89.75 μl of the transfection mix were added per neuronal culture dish. The neuronal cells were incubated for 3 h at 37°C until precipitates were formed. To remove the precipitates, the cells were washed twice with 2 × HBS. Therefore 1 ml prewarmed 2 x HBS was added to the transfected neurons before 1 ml was removed. This step was repeated once and the medium-HBS mix was afterwards removed completely. To provide important growth factors for neuronal growth, 2 ml of the collected old medium were added to each dish. The cells were incubated at 37°C for 18–20 h and analyzed by live cell imaging.

For transient transfection of HEK and CHO cells with different APP695 constructs or the meprin β construct a transfection mixture containing 8 μg polyethylenimine (PEI) and 2 μg DNA in 120 μl serum-free medium was added to the cells for 4 h.

Stable CHO cells were generated as described previously (Isbert et al., 2012) using pLHCX-APP695 K587C and 350 μg/ml Hygromycin B (Thermo Fisher Scientific) for selection.

### Antibodies

The antibody mix 1G75A3 of the two monoclonal antibodies 1G7 and 5A3, both directed against the APP ectodomain, was provided by Dr. Koo (UC San Diego School of Medicine, USA) and enabled the detection of all forms of full-length APP (mature, immature or dimerized) in cell lysates as well as soluble APP in the conditioned medium. This antibody mix was used for Western Blotting and for immunoprecipitation of APP. For detection of LRP1 in Western Blotting the polyclonal antibody 1,704 (Pietrzik et al., 2002) directed against the C-terminus of LRP1 was used. Y188 (Abcam) directed against the C-terminus of APP was used to detect monomeric and dimeric APP in Blue Native Gel Electrophoresis. Aβ was detected by IC16, a monoclonal antibody recognizing the amino acids 1 to 16 of the human Aβ sequence (Jäger et al., 2009). The polyclonal anti-actin antibody and the secondary HRP-conjugated goat anti-rabbit antibody were purchased from Sigma-Aldrich. The secondary donkey antibody against mouse, also HRP-conjugated, was obtained from Jackson ImmunoResearch.

### Western blotting

After collecting the conditioned medium cells were harvested and lysed either in RIPA (50 mM Tris-Cl (pH 8), 150 mM NaCl, 0.1% SDS, 1% Nonidet P-40, 10 mM NaF, 1 mM β-glycerophosphate, 0.5% sodium deoxycholate) regarding neurons or, concerning HEK and CHO cells, in NP-40 lysis buffer (500 mM Tris (pH 7.4), 150 mM NaCl, 5 mM EDTA, 1% Nonidet P-40, 0.02% NaN_3_) both containing 1 x protease inhibitor cocktail (PI; Roche). Debris were pelleted by centrifugation with 18,600 × g for 20 min at 4°C. The protein concentrations were measured using the Pierce™ BCA Protein Assay Kit (Thermo Fisher Scientific) to determine equal amounts of total protein for lysate analysis. For comparable protein amounts of the conditioned media volumes were adjusted to the protein concentration in the corresponding lysates. After addition of 4 x SDS sample buffer with (Roti®-Load 1; Roth) or without (40% glycerol, 200 mM Tris-HCl (pH 6.8), 0.08% bromphenol blue, 8% SDS in VE-H_2_O) β-mercaptoethanol (βME) samples were boiled for 5 min at indicated temperatures. Proteins were separated by gel electrophoresis in 6 or 7% Bis-Tris gels and transferred onto nitrocellulose membranes (GE Healthcare Life Sciences) via wet blot. To block non-specific binding membranes remained for 1 h in 5% (w/v) non-fat dry milk dissolved in TBS containing 0.05% Tween 20 (Roth) before incubation with the appropriate primary and secondary antibodies. The protein detection was carried out using the Immobilon Western HRP Substrate (Millipore) resulting in chemiluminescence, which was recorded by the LAS-3000 mini (Fujifilm).

### Immunoprecipitation and detection of Aβ peptides

The immunoprecipitation of Aβ peptides was performed as described by Schönherr et al. (2016). Proteins were separated by Urea SDS-PAGE corresponding to the approach of Klafki et al. (1996) and transferred to PVDF membranes via semi-dry Western Blotting (Biorad) at 47 mA per gel. Afterwards membranes were boiled for 3 min in 1 x PBS before blocking non-specific binding in 5% (w/v) non-fat dry milk in TBST for 30 min. Membranes were incubated over night at 4°C with IC16 antibody (1:500). After washing with TBST the secondary HRP-cojugated mouse antibody was added for 1 h at room temperature. Protein detection and recording were performed as described above.

### Blue native gel electrophoresis

Blue native gel electrophoresis was performed as described before (Eggert et al., 2009). Briefly, transfected cells were resuspended in 1 ml of homogenization buffer (250 mM sucrose in 20 mM HEPES, pH 7.4, with protease inhibitors) and then sheared by passing through a 27 gauge needl. Postnuclear supernatant was collected after a centrifugation step at 1,000 × g for 15 min. After sedimentation at 100,000 × g for 1 h the membrane fraction was washed once with 200 μl of homogenization buffer followed by another centrifugation at 100,000 × g. The pellet was resuspended in 200 μl homogenization buffer. 100 μg of protein were solubilized with Blue Native sample buffer (1.5 M amino caproic acid, 0.05 M Bis-Tris, 10% *n*-dodedecyl-_-D-maltoside, and protease inhibitor at pH 7). The samples were separated on gradient gels Thyroglobulin (669 kDa), apoferritin (443 kDa), catalase (240 kDa), aldolase (158 kDa), and bovine serum albumin (66 kDa) were used as molecular weight standards.

### Live cell imaging

Fluorophore tagged LRP1 and APP fusion proteins were tracked by imaging of living cells, as described before (Szodorai et al., 2009; Hermey et al., 2015). Briefly, during live cell imaging transfected cells were temperature-controlled (37°C) and CO_2_-controlled (5%). Images were taken every 200 ms over a period of 30 s. GFP-tagged proteins were excited with 470 nm and RFP fusion proteins with 550 nm wave length using a matching filter and fast changing LED's. Kymographs were created using Image J software (1.46r) in combination with the Multiple-Kymograph plugin. The slope of the traces is a direct measure for the velocity of the vesicles (*v* = cotan(α), where α is the angle relative to the x-axis). Single tracks with an angle 0° < α <90° were defined as anterograde, and tracks with a slope 90° < α <180° were defined as retrograde transport vesicles. Tracks with slopes of 90° (parallel to the time axis) were determined as stationary vesicles. For vesicle distribution all lines of one kymograph were counted as individual transport vesicles and the sum of all anterograde, retrograde and stationary vesicles was set to 100% (given as relative amount of vesicles). For calculation of total amount of vesicles per neurite segment, again all traces of individual kymographs were counted as single vesicles (stationary, anterograde and retrograde vesicles) and related to a neurite length of 1 μm.

### Immunocytochemistry

Primary cortical neurons (PCN) were differentiated for 7 days *in vitro* and then subjected for immunocytochemical analysis. PCN were fixed for 10 min at 37°C in 4% (w/v) PFA with 4% (w/v) sucrose and permeabilized for 10 min with 0.1% (v/v) NP40 in 1 x PBS. For detection of LRP1 and APP we used the polyclonal antibody 1,704 and monoclonal antibody C1/6.1, respectively. Secondary antibodies were Alexa Flour 488 and Alexa Flour 594 (1:1,000, Invitrogen). Hoechst (33258, Thermo Fisher Scientific) was used as nuclear counterstaining. Imaging was performed with microscope Axio Observer Z.1 (Zeiss with apotome) and z-stacks were taken in 0.2 μm steps.

### Pulse-chase assay

To examine the expression and stability of APP dimers, a pulse-chase assay was performed with CHO K1 and CHO 13-5-1 cells 48 h after seeding on 6 cm dishes. Cells were starved in DMEM without methionine and cysteine complemented as described above, which was replaced after 1 h by 1 ml of the same medium containing 150 μCi35S/ml (EasyTag™ EXPRESS35S Protein Labeling Mix; PerkinElmer). Following 15 min incubation at 37°C the medium was substituted to 2 ml α-MEM supplemented as outlined above but with addition of 40 mM HEPES (Lonza). Cells were maintained in this medium at 37°C for indicated time spans before being harvested and lysed in NP-40 buffer with 1 x PI as detailed previously. For immunoprecipitation of APP, lysates and conditioned media were incubated over night at 4°C with protein G agarose beads (Roche) and the 1G75A3 antibody mix against the APP ectodomain. Beads were washed as described above, pelleted and finally boiled in 4 x SDS sample buffer at 80°C for 5 min. The accordingly recovered proteins were separated on 4–12% NuPAGE gradient gels (Invitrogen). After electrophoresis gels were incubated in fixation buffer (10% acetic acid and 20% ethanol in VE-H_2_O) for 15 min and washed for 1 h with VE-H_2_O thereby renewing the water every 20 min. Gels were dried onto chromatography paper (Whatman) for 2 h at 65°C using the Model 583 Gel Dryer (Bio-Rad). Exposure of the film was carried out over night at room temperature in an exposition cassette. Radioactivity was detected by a phosphor imager (Cyclone Plus Storage Phosphor System; PerkinElmer) and visualized via the OptiQuant software.

### Tat-Cre treatment

PCN of 5xFAD/*Lrp1*^*flox*/*flox*^ mouse embryos (E14) were treated with Cre-recombinase fused to a basic protein translocation peptide derived from HIV-TAT (Tat-Cre) (provided by Dr. Roosmarijn E. Vandenbroucke; Inflammation Research Center, VIB, Ghent, Belgium; Department of Biomedical Molecular Biology, Ghent University, Ghent, Belgium) at DIV 4. Therefore, the culture medium was reduced to 2 ml and the recombinase was added in a final concentration of 200 nM. As control, PCN were treated with the Tat-Cre buffer (20 mM HEPES, 0.6 M NaCl, pH 7.4). Cells were incubated with the Tat-Cre recombinase or the vehicle alone for 48 h at 37°C before cell lysis at DIV 6.

### Inhibitor treatment

To study the processing of APP dimers at the cell surface CHO cells stably expressing APP695 K587C were treated with ADAM10 inhibitor (GI254023X; provided by Dr. Ludwig, TU Aachen) 24 h after seeding on 6 cm dishes. Therefore, the medium was reduced to 2 ml fresh medium containing 10 μM ADAM10 inhibitor (stock solution: 10 mM in DMSO stored at −20°C) or DMSO as vehicle control. Cells were maintained under these conditions for 24 h.

### Cell surface-biotinylation

The surface levels of APP dimers were examined 24 h after reduction of the medium and inhibitor treatment. After collecting the conditioned medium cells were rinsed 3 times with ice-cold PBS. Surface proteins were biotinylated by addition of 0.25 mg/ml Sulfo-NHS-LC-LC-Biotin (Thermo Fisher Scientific) dissolved in 1 x PBS for 40 min at 4°C thereby refreshing the biotin solution after 20 min. To quench unconjugated biotin, cells were washed 4 times with 50 mM NH_4_Cl in ice-cold 1 x PBS. Cells were lysed in NP-40 buffer containing 1 x PI. Equal protein amounts were incubated over night at 4°C with NeutrAvidin Agarose Resin (Pierce). Unbound proteins were removed in 3 washing steps with NP-40 buffer and centrifugation at 4°C with 24 × g for 2 min. Beads were boiled at 80°C in 4 x SDS sample buffer for 5 min to elute proteins, which were separated on 6% Bis-Tris gels.

### CSF isolation

Cerebrospinal fluid (CSF) was harvested from 4 months old 5xFAD/*Lrp1*^*flox*/*flox*^ and 5xFAD/*Lrp1*_BE_^−/−^ mice by puncture of the cisterna magna as described previously (Vandenbroucke et al., 2012; Storck et al., 2016). Cell free CSF was obtained by centrifugation at 800 × g for 10 min at 4°C. 2 μl of CSF were diluted in water and mixed with equal amounts of 2 x loading dye (0.72 M Bis-Tris, 0.32 M Bicine, 30% (w/v) sucrose, 2% SDS, 0.02% bromophenol blue without βME). Samples were denatured at 70°C for 5 min to maintain putative dimerization of sAPP. Samples were separated by SDS-PAGE on 7% polyacrylamide SDS gels, transferred onto nitrocellulose membranes (Amersham Hybond ECL) and then blocked in 5% (w/v) non-fat dry milk in TBST (20 mM Tris, 137 mM NaCl, 0.1% (v/v) Tween-20). The antibody mix 1G75A3 (1:300) was used to detect sAPP.

### Animals

*In vivo* analyses were performed with tamoxifen-inducible 5xFAD mice lacking *Lrp1* in brain endothelial and choroid plexus epithelial cells (5xFAD/*Lrp1*_BE_^−/−^) (described in detail in Storck et al., 2016). 5xFAD mice, which represent a well-established AD model harboring 3 *APP* mutations and 2 *PSEN1* mutations that are linked to FAD, served as LRP1 expressing controls. All animal studies were conducted in compliance with European and German guidelines for the care and use of laboratory animals and were approved by the Central Animal Facility of the University of Mainz and the ethical committee on animal care and use of Rhineland-Palatinate, Germany. Mice were housed on a 12-h-light cycle and had *ad libitum* access to water and a standard laboratory diet. To induce knock-out of *Lrp1* in CSF-secreting epithelial cells of the choroid plexus in 5xFAD/*Lrp1*_BE_^−/−^, 12-week-old animals were injected i.p. with 2 mg tamoxifen (T5648, Sigma-Aldrich) for 7 consecutive days as described in Storck et al. (2016). After tamoxifen injection the standard laboratory diet was changed to chow supplemented with 400 mg tamoxifen citrate per kilogram dry weight (CRE Active TAM400, LASvendi) to maintain Cre-mediated recombination.

### Quantification and statistical analysis

Western Blots and phosphor imager results were quantified by densitometry using ImageJ (1.44 or 1.46r) or Multi Gauge V3.0, respectively. The Graph Pad Prism 4 software (Graph Pad; La Jolla) provided the basis for compilation of the shown graphs and for statistical analysis. Data were analyzed by Student's *t*-test or one-way ANOVA followed by Tukey's *post-hoc* test. For live cell analysis at least 5 kymographs were analyzed. Student's *t*-test was used when comparing only two sets of data or one-way ANOVA followed by Bonferroni *post-hoc* test when comparing three sets of data and given the data were normaly distributed, respectively. The Kruskal-Wallis-Test followed by Dunn's Multiple Comparison Test was used to assess statistical differences between three sets of data given that data weren't normally distributed or variance was significantly different. The level of significance was set at *p* < 0.05 (^*^), *p* < 0.01 (^**^) and *p* < 0.001 (^***^).

## Results

### APP dimers are generated and processed in cortical neurons

**Figure 1:**
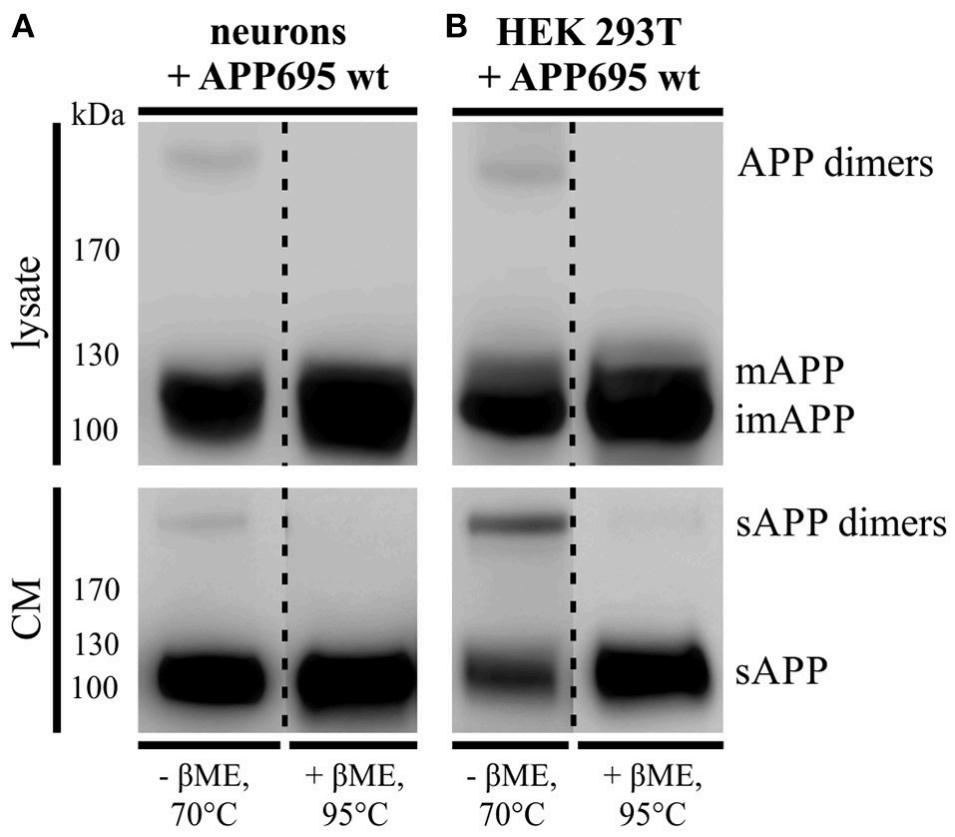
**APP dimer generation and processing takes place in primary cortical neurons. (A)** Murine primary cortical neurons (DIV 7) were infected with an adenoviral vector encoding human APP695 while **(B)** HEK 293T cells were transiently transfected with the pLHCX-APP695 wt construct. 24 h post infection or transfection, respectively, conditioned media (CM) were collected and cells were lysed in RIPA (PCN) or NP-40 (HEK) lysis buffer. Via the antibody mix 1G75A3 (1:3,000) APP was detected in lysates (upper blots) and conditioned media (lower blots). PCN show similar APP dimer expression in the lysate as HEK cells and also generate soluble APP dimers. Under reducing conditions using β-mercaptoethanol (βME) and heating at 95°C the dimer band disappeared. All lanes of lysate or conditioned medium are on the same blot but were rearranged for better presentation.

As described before 30–50% of APP are present in a dimerized form in human brain (Munter et al., 2007; Schmidt et al., 2012). To investigate, whether APP695 dimer formation can be analyzed in a neuronal system, we infected primary cortical neurons of C57BL/6J mouse embryos (E14) with an adenovirus driving expression of human APP695. Indeed, we were able to detect APP dimers in the lysate of DIV 8 mouse neurons (Figure 1A) comparable to the expression of human APP dimers in HEK cells (Figure 1B). Likewise, by analyzing the supernatant of the same cells, we observed soluble APP dimers in the conditioned medium suggesting that APP dimers are not only generated but also processed in neuronal cells as well as in human kidney cells (Figures 1A,B). To verify the existence of APP dimers linked by disulfide bridges as we have described before (Isbert et al., 2012), the samples were boiled in sample buffer containing β-mercaptoethanol (βME). Note, that in the samples under reducing conditions the disulfide bonds were dissolved and the dimer band signal decreased, whereas the signal intensity for monomeric APP increased (Figures 1A,B). These data show that disulfide-bound sAPP dimers, formed most likely in the ER, are anterogradely transported and shed by secretases in a dimerized status.

### Monomeric and dimeric APP show similar transport characteristics

**Figure 2:**
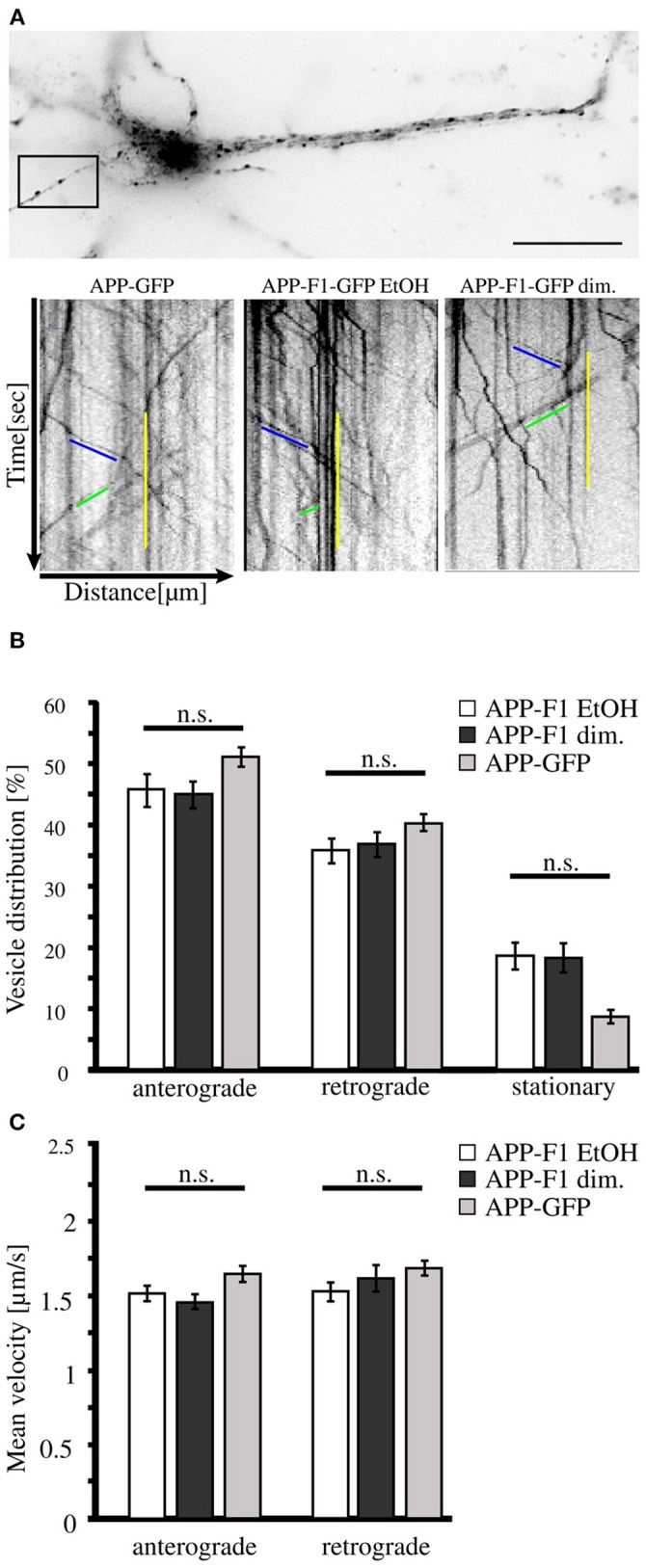
**APP dimerization does not affect its transport characteristics**. Murine cortical primary neurons (DIV 6) were transiently transfected with expression constructs encoding APP-GFP or APP-F1-GFP. After 18–20 h and 1 h prior live cell recording of axonal vesicle movements, APP-F1-GFP expressing neurons were either treated with 100 nM AP20187 (dimerizer) or for control with the vehicle of the dimerizer, ethanol (negative control). **(A)** Representative primary neuron and kymographs of cells expressing APP GFP or APP-F1-GFP treated with dimerizer or ethanol respectively. The ROI is marked by a rectangle. Bar: 20 μm. **(B)** Vesicle distribution and **(C)** anterograde and retrograde transport velocities of APP-GFP, non-dimerized APP-F1-GFP (ethanol control) and APP-F1-GFP dimerized vesicles. No differences among APP variants could be observed (one-way ANOVA followed by Bonferroni post hoc test). Bars represent mean values ± SEM, *n* = 3 (≥16 cells per approach).

As we found that neurons secrete disulfide-bound dimerized sAPP, we wondered if monomeric and dimeric APP are transported along the secretory pathway in the same or diverse types of transport vesicles. For this purpose we used an inducible FK501-binding-protein (FKBP) -based dimerization system (Rollins et al., 2000), previously used for analysis of APP processing in dependence of APP dimerization (Eggert et al., 2009). For live cell imaging expression constructs encoding for C-terminal tagged GFP APP-FKBP fusion proteins were generated (APP-F1-GFP) (Video in Supplementary Material 1). For control, we first verified that APP-GFP and non-dimerized APP-F1-GFP exhibit identical transport characteristics (Figure 2A). Futhermore, as GFP has a weak tendency to self-dimerize (Chalfie and Kain, 2005), we tested if APP-GFP might exhibit in comparison to APP altered dimerization properties, by using the blue-native gel system (Eggert et al., 2009). Notably, we observed for APP-GFP no increase in dimerization properties (Supplementary Figure 1). In the next step, the transport of non-dimerized (APP-F1-GFP + EtOH) and dimerized APP (APP-F1-GFP + dim.) was compared (Figure 2A). Surprisingly, the induction of APP dimerization had no significant influence on APP transport velocities in anterograde or retrograde direction, respectively (Figures 2B,C). The majority of APP vesicles moved with a velocity between 0.5 and 2.5 μm/s in both directions, independent of their dimerization status. These data suggest that monomeric and dimeric APP are transported by the same kinesin dependent transport machinery.

### LRP1 deficiency leads to accelerated trafficking of APP dimers

**Figure 3:**
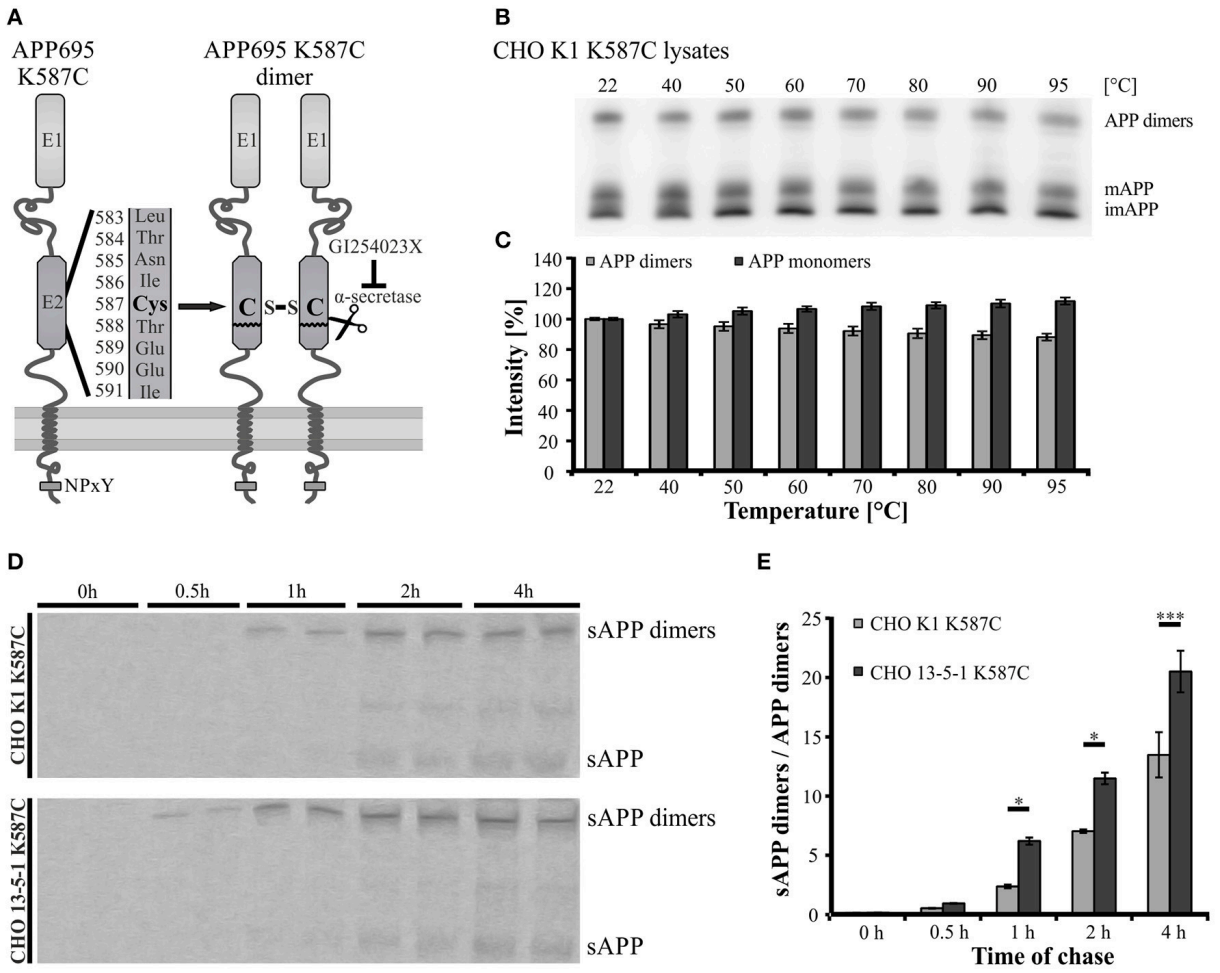
**Faster trafficking of SDS- and heat-stable APP ***cis***-dimers in LRP1-deficient cells. (A)** Schematic representation of the APP Cys-mutant encoded by the pLHCX-APP695 K587C construct and its resulting *cis*-dimerization state. **(B,C)** CHO K1 cells were transiently transfected with the APP695 K587C construct. **(B)** 24 h post transfection monomeric as well as dimerized APP were detected by the antibody mix 1G75A3 (1:3,000) in cell lysates. **(B,C)** Heating of samples up to 95°C shows a negligible reduction of the APP dimer signal with a comparable increase in monomeric APP. **(D)** CHO K1 and LRP1-deficient CHO 13-5-1 cells both stably expressing the dimer bearing APP construct were pulsed with radiolabeled sulfur (^35^S) for 15 min. Chase was performed after stated time spans prior to immunoprecipitation of APP with the antibody mix 1G75A3 (1:300) and SDS-PAGE. Exposure of the film revealed an earlier occurrence of soluble APP dimers in the conditioned medium of LRP1-deficient CHO 13-5-1 cells (30 min chase) than in CHO K1 cells (1 h chase). **(E)** Comparison of the sAPP dimer to APP dimer ratio of both cell types shows a significant elevation for CHO 13-5-1 cells beginning after a 1 h chase. Bars represent mean values ± SEM, *n* = 3; Student's *t*-test; *p* < 0.05 (^*^), *p* < 0.001 (^***^).

Since APP *cis*-homodimers and monomers show similar transport characteristics, we assumed that both follow the same principle. Previously, we demonstrated that LRP1 influences monomeric APP transport along the secretory pathway (Waldron et al., 2008). Therefore, we wanted to analyze now, whether a lack of LRP1 may also affect trafficking of APP dimers. For this purpose, we generated a cDNA construct providing the continuous expression of SDS-stable APP *cis*-dimers. The expression construct exhibits a triplet mutation in the coding sequence of APP695, which leads to an amino acid exchange from lysine (K) to cysteine (C) at position 587 (APP695 K587C) (Figure 3A). This mutation enabled the formation of APP *cis*-dimers by disulfide bridges between the cysteine residues in the E2 domain of two mutant APP molecules. According to our expectation a stepwise increase of temperature up to 95°C showed only a slight decrease of APP K587C dimers, indicating that most of the APP K587C dimers are stabilized by intramoleclular disulfide bonds (Figures 3B,C). To get further insights on the generation and processing of APP *cis*-dimers in regard to LRP1, we performed a pulse-chase assay with CHO K1 and LRP1-deficient CHO 13-5-1 cells stably expressing APP695 K587C dimers. This assay revealed that sAPP dimers were already immunoprecipitated after a 30 min chase in CHO 13-5-1 cells while in CHO K1 cells shed APP dimer fragments were first detectable after a 1 h chase (Figure 3D). Quantification of the sAPP dimer/APP dimer ratio showed an increase of this ratio in LRP1-deficient cells (Figure 3E) suggesting an earlier availability of APP dimers for shedding at the cell surface. Thus, these results point to an accelerated trafficking of APP dimers in the absence of LRP1.

### LRP1 alters APP transport characteristics

We observed that anterograde transport of monomeric and dimeric APP is affected by LRP1, suggesting that LRP1 and APP might be sorted in common transport vesicles and that LRP1 might be required for APP sorting. To address this hypothesis, we performed co-stainings of endogenous APP and LRP1 in primary cortical neurons (PCN) and used again a live cell imaging approach.

Immuncocytochemical analysis of PCN differentiated for 7 days *in vitro* using anti-APP and anti-LRP1 antibodies revealed a strong cytoplasmic staining within the cell body and a punctate staining of LRP1 and APP in neurites, at least in part representing transport vesicles (Supplementary Figure 2). Interestingly, we observed only a low co-localization rate, arguing that LRP1 and APP are mostly transported in different transport vesicle types.

**Figure 4:**
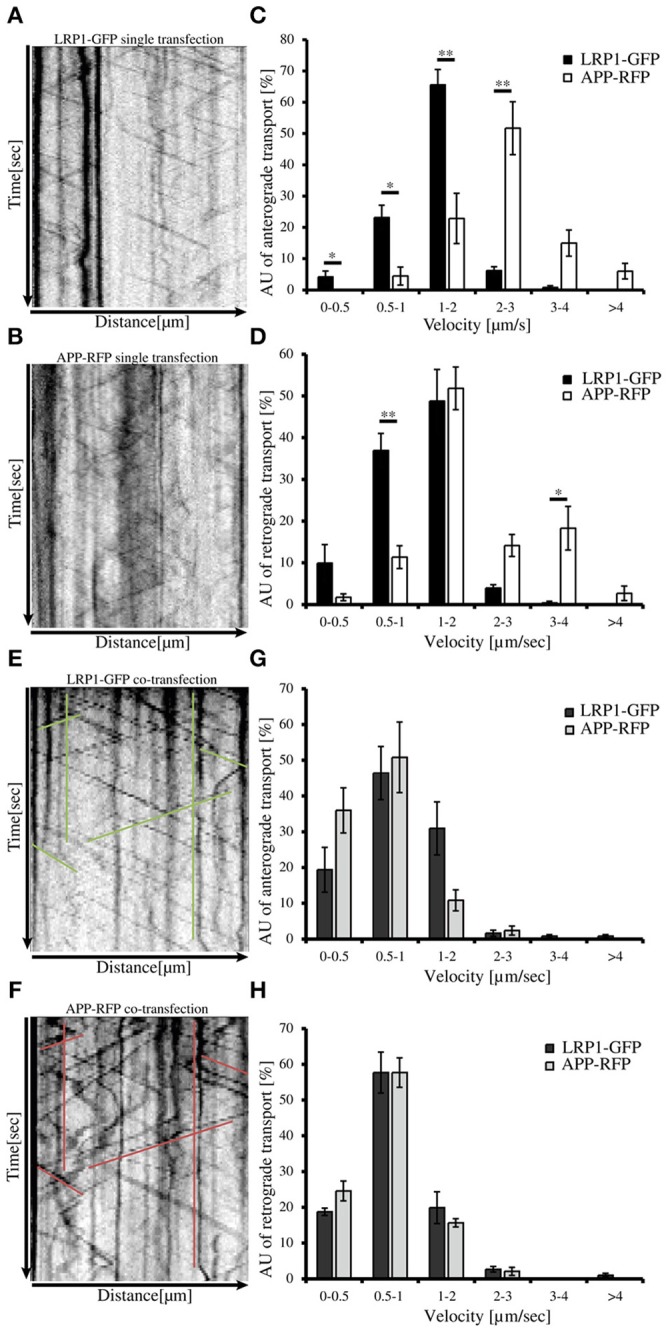
**LRP1 recruits APP in common transport vesicles**. Primary hippocampal neurons from mice (P0) were differentiated for 6 days *in vitro*, expressing either **(A)** only LRP1-GFP, **(B)** APP-RFP or **(E,F)** alternatively co-expressing both, APP-RFP and LRP1-GFP were subjected for live cell imaging 18–20 h post transfection. Time lapse series were plotted as kymographs (representative kymographs, single transfection: **A**,**B**; co-transfection: **E**,**F**) and used for determination of individual transport vesicle velocities. For quantification of transport velocities >5 kymographs from different cells were analyzed [**(C,D)** LRP: *n* = 5 cells, *n* = 534 vesicles; APP: *n* = 7 cells, *n* = 254 vesicles; **(G,H)** *n* = 5 cells, *n* = 371 vesicles]. **(C)** Anterograde and **(D)** retrograde transport vesicles containing APP-RFP (white columns) or LRP-GFP (black columns). Note the change of APP-RFP transport characteristcs in **(G)** anterograde and **(H)** retrograde direction (light gray columns) upon co-expression of **(G,H)** LRP1-GFP (dark gray columns). Bars represent mean values ± SEM, *n* > 5 (≥254 vesicles); Student's *t*-test, *p* < 0.05 (^*^), *p* < 0.01 (^**^).

An expression construct encoding an N-terminal myc tagged LRP-mini-receptor (Rabiej et al., 2015) was used for generation of a C-terminally GFP tagged LRP-mini-receptor (LRP1-GFP). After verification that the newly generated LRP1-GFP fusion protein was expressed as full-length protein and that the GFP-tag did not alter the subcellular localization (data not shown) the construct was used for live cell imaging. First, we wanted to analyze transport velocities of APP-RFP and LRP1-GFP in single transfected primary hippocampal mouse neurons (PHN). Time lapse series of 30 s were recorded at an interval of 200 ms/frame and vesicle movement was quantified based on the analysis of kymographs (Figures 4A,B; Video in Supplementary Material 2). Quantification showed that the largest fraction (68%) of anterograde LRP1-GFP-positive vesicles was transported with a velocity of 1–2 μm/s in contrast to APP-RFP that was mostly (66%) transported in vesicles faster than 2 μm/s (Figure 4C). Also for retrograde moving vesicles, a clear difference in transport characteristics was observed (Figure 4D). Although most of the retrograde transport vesicles containing APP-RFP and LRP1-GFP moved with a velocity of 1–2 μm, a fraction of APP-RFP positive transport vesicles showed retrograde transport characteristics with velocities >2 μm/s, which was not observed for LRP1-GFP containing vesicles. These data suggest that the majority of LRP1 and APP are transported in distinct anterograde transport vesicles, whereas a larger fraction of LRP1 and APP is co-transported in retrograde transport vesicles.

**Figure 5:**
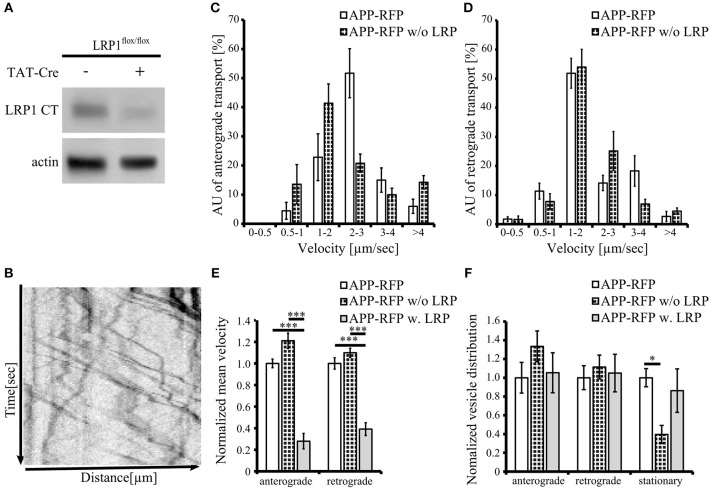
**Loss of LRP1 leads to increased transport vesicles rates**. Primary cortical neurons (PCN) (DIV4) of C57BL/6J wild type or *Lrp1*^*flox*/*flox*^ mouse embryos were treated with 200 nM Tat-Cre recombinase or vehicle control for 48 h. **(A)** Representative blots of PCN from 5xFAD/*LRP1*^*flox*/*flox*^ mouse embryos treated with Tat-Cre showed a decrease in LRP1 CT (1704 antibody) expression by approximately 2-fold compared to vehicle treated control. Anti-actin staining served as loading control. **(B)** 24 h prior live cell imaging analysis, PCN were transiently transfected with cDNA encoding APP-RFP. Time lapse series from live cell imaging were plotted as kymographs (representative kymograph, **B**) and used for determination of individual transport vesicle velocities. **(C)** Anterograde and **(D)** retrograde transport velocity profiles of APP-RFP in wild type (white columns) and LRP1 deficient PCN (black white columns). Normalized transport velocities **(E)** and relative distribution **(F)** of APP-RFP in wild type PCN (white columns), LRP1 deficient PCN (black white columns) and PCN co-expressing LRP1. Note the lower amount of stationary vesicles in LRP1 deficient neurons. For quantification of transport velocities >5 kymographs from different cells were analyzed (APP-RFP in neurons of C57BL/6J mice: *n* = 7 cells, *n* = 254 vesicles; APP-RFP in LRP1 deficient neurons: *n* = 6 cells, *n* = 573 vesicles; APP-RFP in LRP1-GFP co-expressing neurons: *n* = 5 cells, *n* = 371 vesicles). Bars represent mean values ± SEM, *n* > 5 (≥254 vesicles); **(C,D)** Student's *t*-test, **(E,F)** Kruskal-Wallis-Test followed by Dunn's Multiple Comparison Test, *p* < 0.05 (^*^), *p* < 0.001 (^***^).

Further, we tested if LRP1 and APP co-expression might affect APP transport characteristics and vice versa. For this purpose, we performed live cell imaging analyses of PHNs co-expressing LRP1-GFP and APP-RFP 18 to 20 h post transfection, as described above (representative kymographs Figures 4E,F). Quantification revealed that LRP1-GFP and APP-RFP are co-transported in common anterograde and retrograde transport vesicles (Figures 4G,H). Most interestingly, co-expression of LRP1-GFP caused a change of APP-RFP transport characteristics, that was highly similar to those observed in single transfected cells for LRP1-GFP (Figures 4A–D), whereas LRP1-GFP transport upon co-expression of APP remained unchanged (Figures 4G,H). This holds true for APP/LRP1 anterograde (Figure 4G) as well as retrograde (Figure 4H) transport. Accordingly, also the mean velocities were strongly reduced upon co-expression of LRP1 (Figure 5E). Notably, the relative amount of anterograde, retrograde and stationary vesicles remained unchanged, arguing that LRP1 not simply holds back APP in the Golgi. Instead, our data indicate that co-expression of LRP1 may cause a recruitment of APP into common transport vesicles, that exhibit different transport characteristics.

To further validate that LRP1 modifies APP intraneuronal transport, we analyzed APP-RFP transport in primary neurons with reduced LRP1 levels. For this purpose, we used PCN of *Lrp1*^*flox*/*flox*^ mouse embryos, treated with 200 nM Cre-recombinase fused to a basic protein translocation peptide derived from HIV-TAT (Tat-Cre) for 48 h prior live cell imaging. Reduced LRP1 expression of about 2-fold was validated by Western Blot analysis (Figures 5A, **7A**). Interestingly, we observed in contrast to LRP1 co-expression only a tendency toward increased APP transport velocity in anterograde direction (*p* = 0.051) (Figures 5C,F) and no change in retrograde direction or for the amount of stationary vesicles (Figures 5D,F). In contrast, LRP1 deficiency caused a significant (*p* = 0.011) decrease of stationary and an increase (*p* = 0.011) of moving transport vesicles (Figure 5F). Seperation of moving vesicle data into anterograde and retrograde transport revealed due to lower n-number not the significance levels (*p* = 0.06) (Figure 5F).

Together, our data show that elevated LRP1 expression causes a decrease of APP transport rate whereas reduced levels of LRP1 cause an increase of APP transport rates.

### LRP1 expression affects processing of APP695 K587C dimers

**Figure 6:**
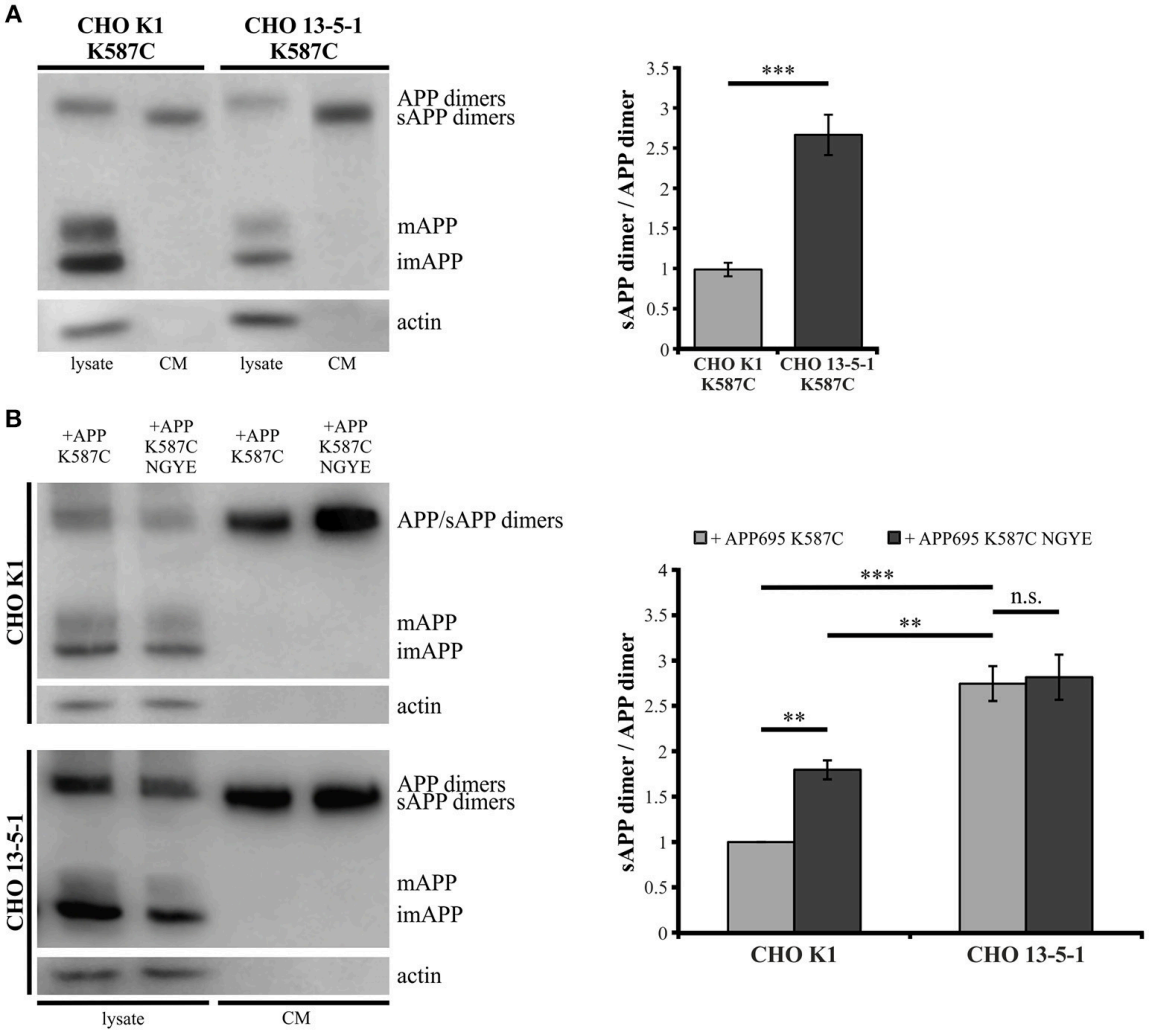
**Reduced internalization of APP dimers leads to increased sAPP dimer generation**. Lysates and conditioned media were probed (24 h post transfection, **B**) with antibodies specific for APP (1G75A3 antibody mix, 1:3,000) or actin (1:1,000). LRP1-deficiency in CHO cells **(A)** stably or **(B)** transiently transfected with pLHCX-APP695 K587C lead to increased sAPP dimer production. **(A)** The ratio of sAPP dimers to APP dimers is significantly increased in CHO 13-5-1 cells (*n* = 7) compared to CHO K1 cells (*n* = 5). Bars represent mean values ± SEM; Student's *t*-test; *p* < 0.05 (^*^), *p* < 0.01 (^**^), *p* < 0.001 (^***^). **(B)** Expression of the APP dimer construct additionally exhibiting the NGYE mutation in the APP internalization motif partially mimicked the LRP1 deficiency in CHO K1 cells while sAPP dimer secretion in CHO 13-5-1 cells remained unaffected. Note that sAPP dimer to APP dimer ratio increased significantly in CHO K1 cells expressing APP695 K587C NGYE compared to those transfected with APP695 K587C. Bars represent mean values ± SEM, *n* = 4; one-way ANOVA with Tukey's *post-hoc* test; *p* < 0.01 (^**^), *p* < 0.001 (^***^).

Showing that the expression of LRP1 alters trafficking of monomeric as well as dimerized APP, we assumed that LRP1 may also affect processing of APP dimers. We previously demonstrated that internalization of APP (mostly monomeric and possibly also dimeric) from the cell surface is reduced in the absence of LRP1 resulting in an increase in sAPPα secretion (Pietrzik et al., 2002). To investigate, whether a similar effect is obtained also for covalently bound APP homodimers, we used CHO K1 and LRP1-deficient CHO 13-5-1 cells both expressing APP695 K587C exogenously. In Western Blot analyses we first compared APP dimer expression and sAPP dimer secretion of both cell lines (Figure 6A). Here, we detected lower APP dimer expression in the lysate of CHO 13-5-1 cells compared to CHO K1 despite a comparable total protein load. However, the ratio of sAPP dimers to dimeric APP of LRP1-deficient CHO cells was approximately 3-fold stronger than in CHO K1 cells. To test, if this difference may be explained by increased processing due to decreased internalization of APP dimers from the cell surface in LRP1-deficient CHO 13-5-1 cells, we transfected CHO K1 and CHO 13-5-1 cells with an APP695 dimer construct, exhibiting a mutation in the APP internalization motif “YENPTY” (Lai et al., 1995; MarquezSterling et al., 1997). The amino acid exchange from NPTY to NGYE leads to a reduced internalization of APP dimers from the cell surface mimicking LRP1 deficiency. We expected that exogenous expression of APP695 K587C NGYE compared to APP695 K587C should increase sAPP dimer secretion in CHO K1 cells, whereas expression in CHO 13-5-1 cells, that already show reduced APP dimer internalization due to the absence of LRP1, should cause no further increase in sAPP secretion. According to our expectation, we detected higher amounts of soluble APP dimers in the conditioned medium of CHO K1 cells, but no significant difference in sAPP dimer secretion in CHO 13-5-1 cells (Figure 6B). This further supports the hypothesis that LRP1 affects internalization of APP monomers and dimers in a comparable manner.

### *Lrp1* knock-out in PCN affects APP dimer processing

**Figure 7:**
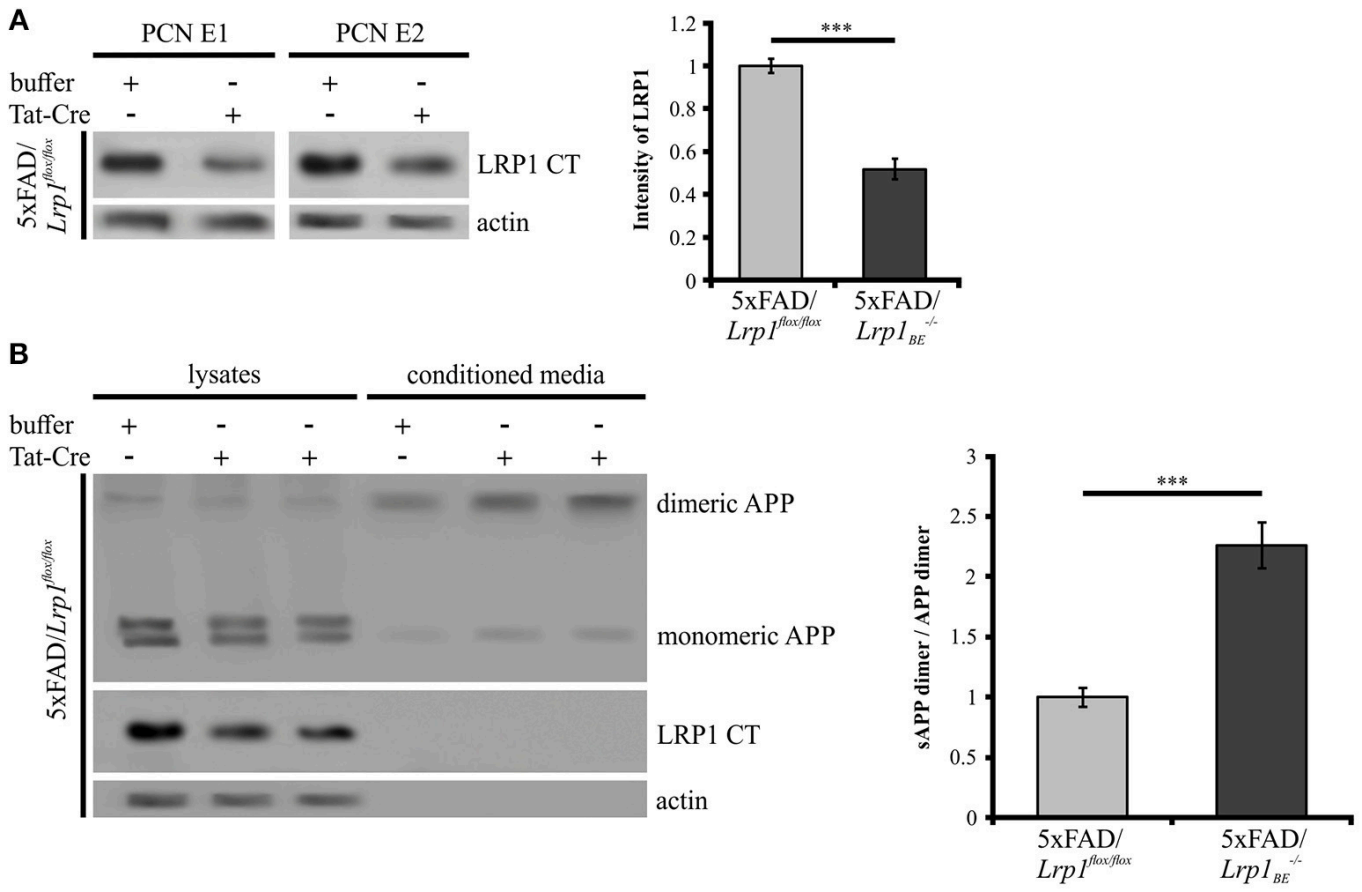
**Partial ***LRP1*** knock-out affects APP processing in PCN**. PCN of 5xFAD/*LRP1*^*flox*/*flox*^ mouse embryos were treated with either 200 nM Tat-Cre or vehicle for 48 h. Lysates and conditioned media were probed with specific antibodies for APP (1G75A3 antibody mix, 1:300), for LRP1 CT (1704 antibody, 1:10,000) and actin (actin antibody, 1:1,000). **(A)** Representative blots of PCN from two different 5xFAD/*LRP1*^*flox*/*flox*^ mouse embryos (E1 and E2) treated with Tat-Cre showed a decrease in LRP1 CT expression by approximately 2-fold compared to vehicle treated controls (normalized to actin). Bars represent mean values ± SEM, *n* = 6; Student's *t*-test; *p* < 0.001 (^***^). **(B)** Representative blot showing a slight APP decrease in lysates and an increase of soluble APP fragments in the conditioned medium of Tat-Cre treated PCN. Partial *Lrp1* knock-out resulted in an increased sAPP dimer to APP dimer ratio by more than 2-fold in comparison to buffer treated PCN. Bars represent mean values ± SEM, *n* = 5; Student's *t*-test; *p* < 0.001 (^***^).

As we could show that APP dimers are formed and processed in primary cortical neurons (Figure 1), we wanted to analyze the effect of a *Lrp1* knock-out on APP dimer processing in neuronal cells. Hence, PCN from 5xFAD/*Lrp1*^*flox*/*flox*^ mouse embryos (E14) were treated with Tat-Cre recombinase to induce the excision of *Lrp1* via recombination of the loxP recognition sites flanking this gene. Western Blot analysis revealed a 2-fold reduction of LRP1 expression in PCN treated with Tat-Cre for 48 h compared to neurons incubated with the vehicle (Figure 7A). We assume that the incomplete reduction of LRP1 was due to its long half-life (24 h) (Reekmans et al., 2010). Interestingly, the sAPP dimer/APP dimer ratio of neurons with a partial *Lrp1* knock-out showed a more than 2-fold increase in comparison to the buffer treated PCN (Figure 7B). These observations are similar to the effects seen in LRP1-deficient CHO 13-5-1 cells and might be explained by a faster transport rate of APP dimers to and/or less internalization from the cell surface. This may result in an elevated APP processing by the active secretases at this site due to earlier and/or prolonged substrate availability.

### Processing of APP *Cis*-dimers by meprin β and ADAM10

**Figure 8:**
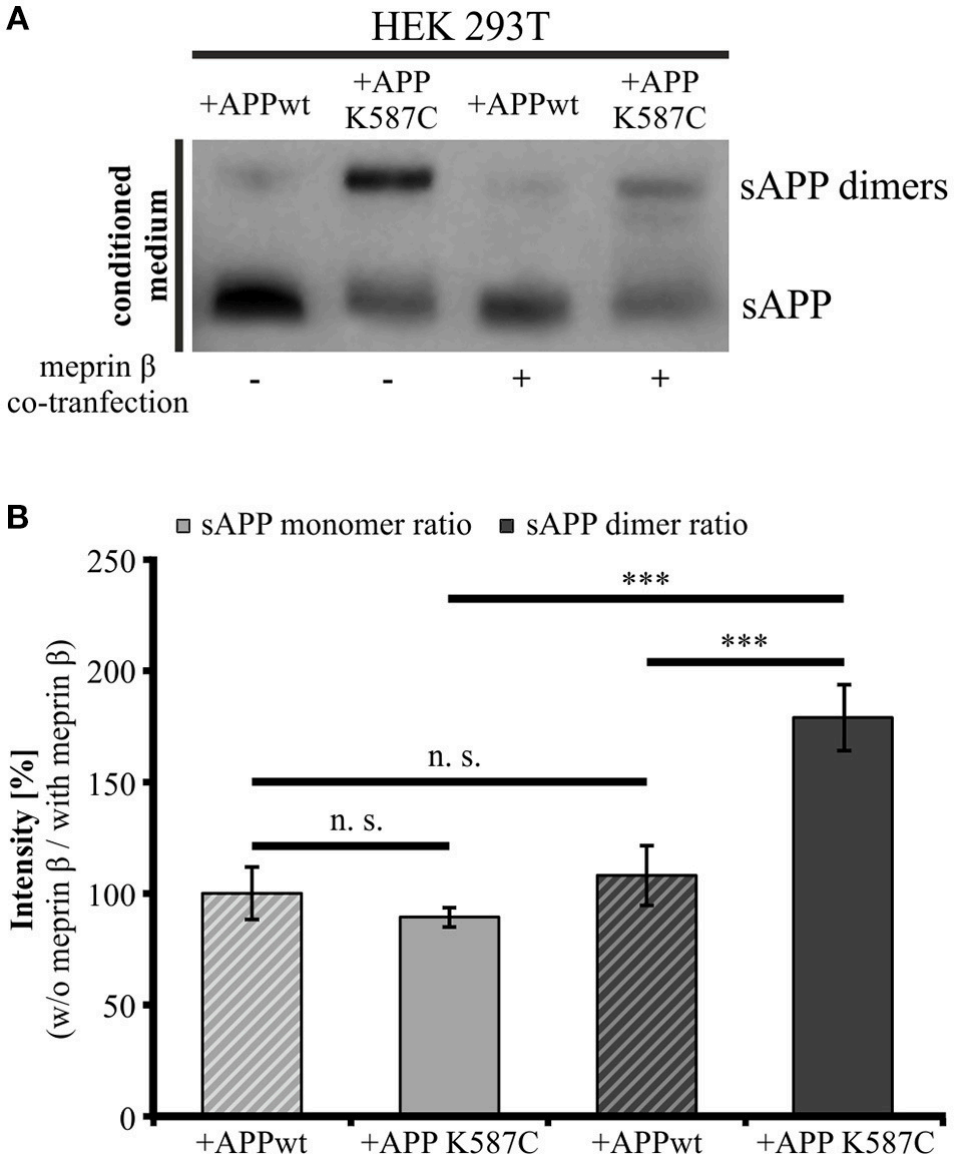
**Meprin β cleaves sAPP dimers with a higher affinity than monomeric sAPP**. HEK 293T cells were transiently transfected with the APP695 wt or the APP695 K587C construct either alone or in co-transfection with meprin β. **(A)** Conditioned medium was probed 24 h post transfection with the antibody mix 1G75A3 (1:3,000) directed against the APP ectodomain. Meprin β expression resulted in a reduced signal intensity of soluble APP, especially prominent for sAPP dimers in cells expressing the APP Cys-mutant (APP695 K587C). **(B)** For quantification the ratio of sAPP monomers and sAPP dimers, respectively, was calculated as the quotient of signal intensities in cells just transfected with an APP construct (w/o meprin β) to appropriate cells co-expressing meprin β (with meprin β). This analysis revealed a similar reduction in dimerized and monomeric sAPP for cells transfected with APP695 wt. In contrast, HEK cells expressing the dimer bearing construct show a significant increase in the sAPP dimer ratio compared to sAPP monomer ratio. Bars represent mean values ± SEM, *n* = 4; one-way ANOVA with Tukey's *post-hoc* test; *p* < 0.001 (^***^).

The presence of soluble APP dimers indicates that APP *cis*-dimers are enzymatically cleaved thereby releasing soluble dimerized fragments. Thus, we wanted to investigate, whether processing of APP *cis*-dimers can be attributed to the same secretases known to be responsible for cleavage of monomeric APP. Regarding the processing of monomeric APP at the cell surface, the metalloproteinases ADAM10 (Weidemann et al., 1989; Lammich et al., 1999) and meprin β (Jefferson et al., 2011; Bien et al., 2012; Schönherr et al., 2016) are implicated. As the metalloproteinase meprin β itself occurs in a dimerized form (Bertenshaw et al., 2003; Kruse et al., 2004), we first focused on the role of meprin β in APP dimer cleavage. To address this point, we co-transfected HEK 293T cells with either APP695 K587C or APP695 wt and the meprin β construct to analyze processing of APP *cis*-dimers in comparison to monomeric APP cleavage by this secretase. As a control for meprin β activity, cells were solely transfected with the wt APP or the dimer-bearing APP construct. As expected, analysis of the conditioned medium of transfected HEK 293T cells revealed higher sAPP dimer levels in cells expressing the dimer-bearing APP construct, compared to those transfected with wildtype APP695 (Figure 8A). Co-transfection with meprin β resulted in a decrease in the signal for monomeric as well as dimerized soluble APP irrespective of the APP construct used for transfection. Interestingly, the reduction of sAPP dimers was considerably stronger than that of monomeric sAPP. To quantify these observations, we calculated the ratio of sAPP dimers secreted from APP/meprin β expressing cells to sAPP dimer secretion of solely APP expressing cells as well as APP K587C/meprin β to APP K587C expressing cells. APP695 wt expressing cells showed no significant difference in the ratio for monomeric and dimerized sAPP, possibly due to the weak signal for sAPP dimers (Figures 8A,B). In contrast, processing of APP695 K587C by meprin β was significantly increased compared to cleavage of monomeric sAPP (Figures 8A,B). In line with this, meprin β co-transfection resulted also in an increase of Aβ secretion (Supplementary Figure 3). Together, these data suggest a higher affinity of meprin β for dimerized than for monomeric APP695.

**Figure 9:**
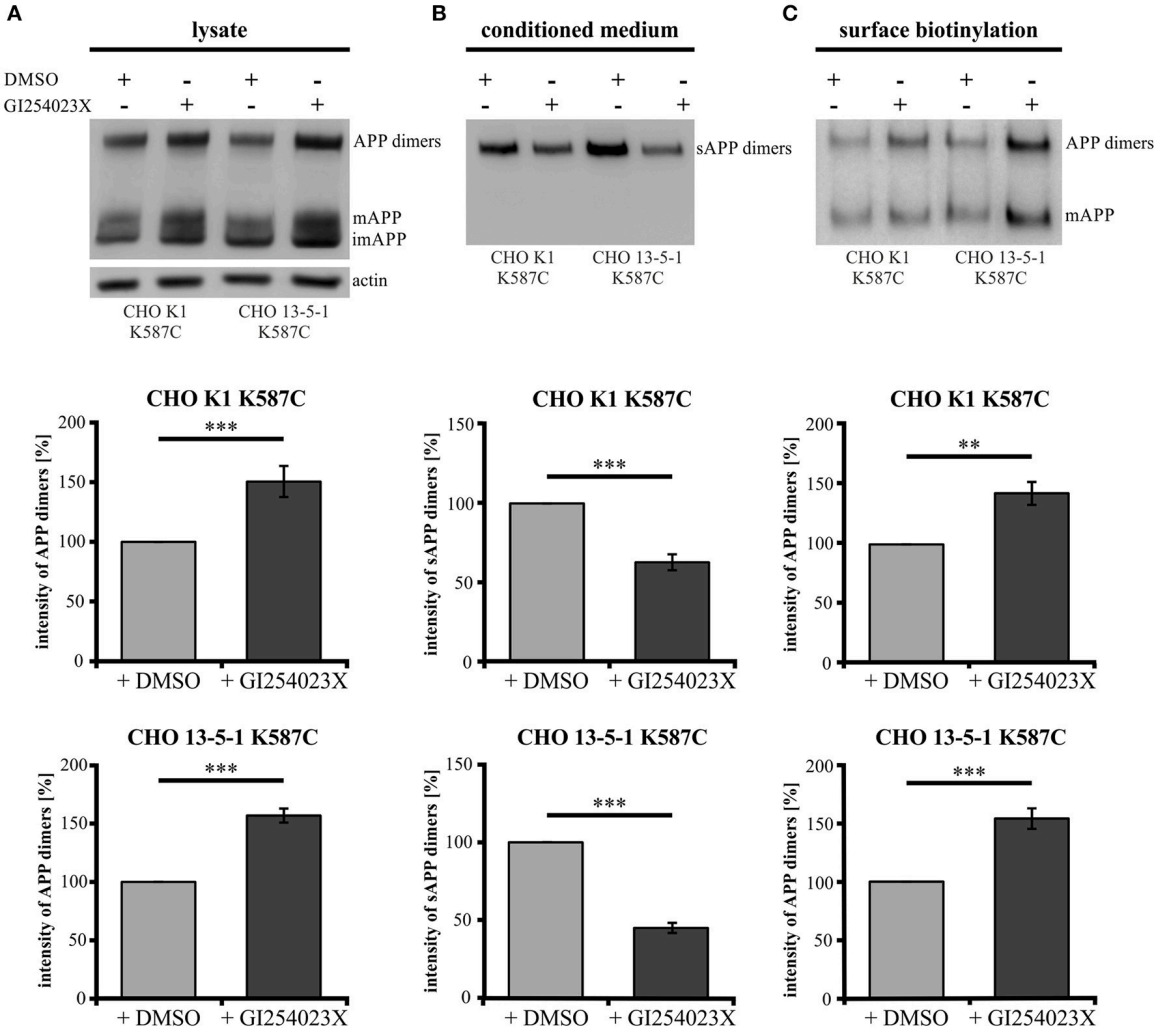
**LRP1 deficiency affects APP dimer processing at the cell surface by ADAM10**. Treatment of APP695 K587C-stable CHO cells (CHO K1 and LRP1-deficient CHO 13-5-1) with the ADAM10-inhibiting compound GI254023X or DMSO (control) was performed 24 h prior to surface biotinylation (sbio) and analysis of lysates and conditioned media (CM) with the APP specific antibody mix 1G75A3 (1:3,000) or the actin antibody (1:1,000), respectively. Bars represent mean values ± SEM, Student's *t*-test; *p* < 0.01 (^**^), *p* < 0.001 (^***^). **(A)** Inhibition of ADAM10 resulted in an increase of APP dimers in the lysate by 50% for CHO K1 (*n* = 7) and by 56% for CHO 13-5-1 (*n* = 7) cells compared to the corresponding DMSO controls. **(B)** In the conditioned medium the decrease of sAPP dimers by ADAM10 inhibition in comparison to DMSO treatment was more substantial in LRP1-deficient cells (55%; *n* = 7) than in CHO K1 cells (38%; *n* = 7). **(C)** The inhibitory effect of the GI254023X compound resulted in an elevation comparing surface expression of APP dimers to DMSO controls. This increase amounted to 43% for CHO K1 and 54% for CHO 13-5-1 cells.

To investigate the role of α-secretase cleavage in APP *cis*-dimer processing, CHO K1 and CHO 13-5-1 cells expressing APP695 K587C were treated with the ADAM10 inhibitor GI254023X (Ludwig et al., 2005). Quantification of APP dimer expression in the lysates of CHO K1 and CHO 13-5-1 cells after incubation with GI254023X revealed an increase of APP dimers of 50 or 56%, respectively (Figure 9A). In line with this, we detected an average decrease in sAPP dimer secretion of 38% for inhibitor treated CHO K1 cells compared to those incubated with the vehicle DMSO alone (Figure 9B). A similar result (55% reduction of sAPP dimers after ADAM10 inhibition) was observed in CHO 13-5-1 cells (Figure 9B). As ADAM10 cleaves APP at the cell surface (Lammich et al., 1999), we expected an accumulation of the mature cell surface exposed APP, after treatment with GI254023X. Indeed, cell surface biotinylation assays using APP695 K587C expressing CHO cells revealed after ADAM10 inhibition an increase of mature cell surface APP dimers of 43% in comparison to DMSO controls (Figure 9C). In CHO 13-5-1 cells treated with GI254023X the surface expression of APP dimers was increased by 54% compared to DMSO controls (Figure 9C), underlining our assumption that LRP1 deficiency accelerates availability of APP dimers for processing by ADAM10.

Together, these data indicate that processing of APP *cis*-dimers can be attributed to the same secretases (meprin β and ADAM10) with meprin β shedding APP preferentially in the dimeric form.

### LRP1 expression affects sAPP dimer secretion *In vivo*

**Figure 10:**
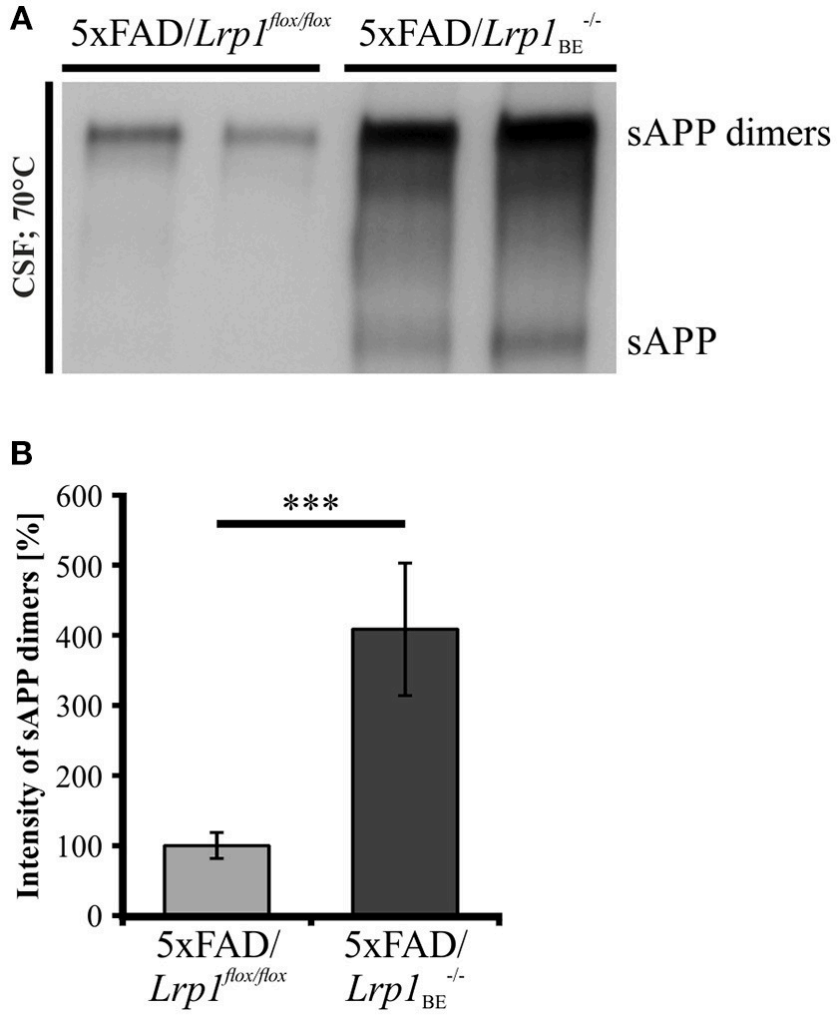
**sAPP dimers in CSF of 5xFAD/***Lrp1***^***flox*****/*****flox***^ and 5xFAD/***Lrp1***_**BE**_^**−/−**^ mice. (A)** Representative Western Blot of 2 μl CSF from 5xFAD/*Lrp1*^*flox*/*flox*^ and 5xFAD/*Lrp1*_BE_^−/−^ mice. Immunostaining with the 1G75A3 antibody mix (1:3,000) revealed **(B)** an about 4-fold stronger immunoreactivity for sAPP dimers in the CSF of 5xFAD mice exhibiting an *Lrp1* knock-out in brain endothelial and choroid plexus epithelial cells (*n* = 3) than in 5xFAD mice expressing LRP1 (*n* = 5). *p* < 0.001 (^***^).

As secretion of monomeric sAPP fragments into the cerebrospinal fluid (CSF) of AD patients has been shown previously (Van Nostrand et al., 1992; Sennvik et al., 2000; Olsson et al., 2003; Brinkmalm et al., 2013), we wanted to study the generation of dimeric sAPP *in vivo*. To investigate, whether LRP1 expression also affects processing of APP dimers *in vivo*, we analyzed the cerebrospinal fluid of 5xFAD mice expressing LRP1 and of 5xFAD mice with a tissue-specific *Lrp1* knock-out in brain endothelial cells and the choroid plexus epithelial cells. In 5xFAD mice only very little amounts of sAPP dimers could be detected (Figure 10A). However, in 5xFAD mice lacking LRP1 in CSF-secreting epithelial cells of the choroid plexus an about 4-fold stronger immunoreactivity for sAPP dimers was observed (Figure 10B). In line with our previous results showing preferred clavage of APP dimers by meprin β (Figure 8), the immense increase in dimerized APP fragments may be explained by the involvement of meprin β besides ADAM10 in APP cleavage at the surface of epithelial cells. These *in vivo* data underline that LRP1 preferentially affects sAPP dimer secretion.

## Discussion

Our data show that LRP1 recruits APP into common fast axonal transport (FAT) membrane bound organelles (MBOs), suggesting that LRP1 functions as a sorting receptor. Thereby, increased levels of LRP1 slow down APP anterograde transport and decrease its endocytosis rate. This in turn causes an increase of surface APP and thus accelerates secretion of sAPP. Interestingly, we observed the same influence for APP monomers and dimers. However, *Lrp1* knock-out in choroid plexus cells increased sAPP monomer secretion, but much more pronounced sAPP dimer secretion in the CSF. This is likely explained by different processing properties of cell surface APP monomers/dimers, as we found that meprin β preferentially cleaves APP dimers.

Our live cell imaging analyses in primary neurons show that LRP1 is anterogradely transported with a median velocity of 1–2 μm/s, (Figure 4), indicating that LRP1 anterograde transport is mediated by the fast axonal kinesin dependent transport (FAT) machinery. Time lapse analysis of APP from our group and others revealed transport velocities of 2–10 μm/s (Figures 2, 4; Kaether et al., 2000; Szodorai et al., 2009; Hermey et al., 2015). Those types of transport vesicles with velocities above 2 μm/s have only been observed very rarely for LRP1 positive vesicles. In line with the low extend of co-localization of LRP1 and APP in neurites, these data indicate that APP and LRP1 are transported in distinct membrane bound organelles (MBO), associated with different FAT machineries. Interestingly, reduced levels of LRP1 in primary neurons caused an increase of APP transport vesicles (Figure 5), whereas co-expression of LRP1 and APP caused an approximation of both transport characteristics, changing APP transport toward velocities observed for LRP1. These data corroborate our previous assumption that LRP1 causes a sorting of APP into LRP1 bearing MBOs (Waldron et al., 2008). As monomeric and dimeric APP are transported with very similar transport characteristics (Figure 2), and as a knock-out of *Lrp1* caused an increase of monomeric as well as dimeric sAPP (Waldron et al., 2008; Figure 3) we assume that LRP1 affects monomeric and dimeric APP in a similar way. In contrast, other sorting receptors of APP, such as SorLA are assumed to affect the equilibrium of APP dimerization, causing different processing kinetics of monomeric and dimeric APP, as indicated by elegant mathematical modeling (Schmidt et al., 2012). Notably, these analyses were performed in cells lacking LRP1. Thus, it would be interesting for future studies to investigate the interplay of LRP1, SorLA and APP dimerization in more detail.

Since LRP1 recruits APP to transport vesicles and the velocity of vesicles carrying APP dimers is similar to APP monomer carrying vesicles we wondered whether APP dimers are released in a similar LRP1 dependent manner as APP monomers (Waldron et al., 2008). In a pulse-chase analysis of LRP1 expressing CHO K1 and LRP1-deficient CHO 13-5-1 cells both stably expressing APP dimers, we detected faster sAPP dimer release in LRP1-deficient cells (Figure 3D). As APP dimerization already takes place in the ER and as those dimers remain stable throughout their transport to the plasma membrane (Isbert et al., 2012; Khalifa et al., 2012), an interaction of dimeric APP with LRP1 early in the secretory pathway may lead to a decelerated APP dimer trafficking, similar as shown for monomeric APP (Waldron et al., 2008). Thus, cell surface processing may be affected, resulting in the delayed occurrence of dimerized sAPP in LRP1 expressing cells.

The interaction of APP with LRP1 also plays an important role at the cell surface as monomeric APP is internalized in a complex with LRP1 by clathrin-mediated endocytosis, thereby affecting its processing (Knauer et al., 1996; Ulery et al., 2000; Pietrzik et al., 2002, 2004; Cam et al., 2005). Hence, we estimated that the accelerated generation of soluble APP dimers in LRP1 deficient cells compared to LRP1 expressing cells (Figure 6A) may result from a reduced APP dimer internalization due to LRP1 deficiency that in turn causes higher APP levels for processing at the cell surface. This assumption is strengthened by the fact that APP dimer constructs, harboring a mutated internalization motif caused an increase in sAPP levels (Figure 6B), as shown before for internalization deficient monomeric APP (Perez et al., 1999). Importantly, the sAPP dimer levels of the internalization deficient mutant were not increased in LRP1 lacking cells. To investigate, whether the LRP1 regulatory effects are similar in neuronal cells, we analyzed PCN with a knock-out of *Lrp1* by Cre recombination. Similar as shown for LRP1-deficient CHO 13-5-1 cells we observed a decrease of APP in the lysates accompanied by an accelerated sAPP generation for monomeric as well as dimeric APP (Figure 7), when LRP1 expression was knocked out in primary neurons. The more than 2-fold increase of the sAPP dimer to APP dimer ratio further supports our hypothesis that LRP1 regulated effects on APP dimer transport and internalization take place in neuronal cells. A resulting earlier and/or prolonged availability of APP may therefore provide more substrate for the cell surface-active shaddases ADAM10 and meprin β. Thus, our data strongly suggest that monomeric and dimeric APP trafficking is equally affected by LRP1 (Ulery et al., 2000; Pietrzik et al., 2002; Cam et al., 2005) and that this process is also important in the neuronal system.

Due to the similar characteristics of monomeric and dimeric APP transport we assumed that APP dimers might also be processed by the same secretases as monomeric APP. Here, we concentrated on cell surface APP. It has been well documented over the last decade that ADAM10 is the most prominent sheddase of APP at the cell surface (Weidemann et al., 1989; Lammich et al., 1999; Jorissen et al., 2010; Kuhn et al., 2010). Recently, the metalloproteinase meprin β was identified to be also capable to process APP at the cell surface (Jefferson et al., 2011; Bien et al., 2012; Schönherr et al., 2016). Here, we show that meprin β as well as ADAM10 are implicated in dimer processing (Figures 8, 9). Inhibition of ADAM10 resulted in a surface accumulation of APP dimers as shown by protein surface biotinylation. This was accompanied by an increase of APP dimers in the lysate and a decrease in soluble APP dimers in the conditioned medium of the tested cells (Figures 9A–C) as shown for monomeric sAPPα (Woods and Padmanabhan, 2013). The role of LRP1 in APP dimer processing is again highlighted by ADAM10 inhibition in LRP1-deficient cells as the demonstrated effects (in CHO K1 cells) were considerably stronger in CHO 13-5-1 cells (Figures 9A–C).

To investigate the role of meprin β in cleavage of APP dimers, we analyzed the sAPP monomer and dimer ratios of HEK cells transfected with APP alone to cells co-expressing meprin β. Interestingly, the sAPP dimer ratio was significantly increased compared to the sAPP monomer ratio (Figure 8) when the stabilized APP homodimer was generated. This indicates that meprin β processes APP, with a preference for APP dimers. Although we do not understand the underlying molecular mechanism yet, it appears reasonable that the higher affinity of meprin β for dimeric vs. monomeric APP is connected to the fact that this secretase itself exists in form of a dimer (Bertenshaw et al., 2003; Kruse et al., 2004). Thus, dimerized APP may offer a cooperative effect on enzymatic activity of meprin β, as recently postulated for α- and β-secretase for APP dimer processing (Schmidt et al., 2012).

Due to the fact that a large fraction of APP occurs in a dimerized form in the brain (Munter et al., 2007; Schmidt et al., 2012) and that APP dimers are processed in PCN of C57BL/6J mice (Figures 1, 7), we asked whether the regulatory effect of LRP1 in APP dimer processing does also play a role *in vivo*. Therefore, we analyzed the CSF of 5xFAD mice and 5xFAD mice with an induced *Lrp1* knock-out in brain endothelial and choroid plexus epithelial cells. Indeed, we were able to detect sAPP dimers to an about 4-fold greater extent in the CSF of mice with the tissue-specific *Lrp1* knock-out than of 5xFAD control littermates (Figures 10A,B). As the choroid plexus epithelial cells express APP (Kalaria et al., 1996; Bergen et al., 2015) and are the main producers of CSF (Brown et al., 2004), sAPP dimers in the CSF presumably originate from these cells. Thus, the increased amount of sAPP dimers in the CSF of 5xFAD/*Lrp1*_BE_^−/−^ mice is likely due to reduced internalization of APP from the surface of the choroid plexus epithelial cells. This may provide more APP dimers for enzymatic cleavage at the cell surface. We could show that ADAM10, the main sheddase of APP at the cell surface (Weidemann et al., 1989; Lammich et al., 1999; Jorissen et al., 2010; Kuhn et al., 2010), is aslo implicated in the cleavage of APP dimers (Figure 9). Thus, the processing of APP dimers in epithelial cells of the choroid plexus may be at least partially performed by this secretase. Furthermore, the especially high levels of sAPP dimers may point to a prominent role of meprin β in this context as we could show that this protease exhibits a preferred activity for dimeric vs. monomeric APP (Figure 8).

Altogether, our studies show that LRP1 affects trafficking of APP monomers and dimers and that APP dimers are preferentially cleaved by ADAM10 and meprin β. Hence, dimerized APP may affect physiological as well as pathogenic functions of APP by different transport and processing characteristics and should be included in future studies regarding the interplay with other sorting receptors than LRP1 or the generation of Aβ species.

## Author contributions

CP and SK conceived and supervised the study. UH, PS, CP, and SK designed the experiments. UH, PS, SES, CT, VR, AJ, SS, NS, CD, and SE performed experiments. UH, PS, SES, CP, and SK analyzed the data. UH and PS wrote and revised the manuscript. CP and SK reviewed the manuscript.

## Funding

Research in our laboratories was funded by the Deutsche Forschungsgemeinschaft (FOR1332, to CP and SK; PI379/6-2, KI819/6-2) and the Stiftung Rheinland-Pfalz für Innovation (to CP and SK).

### Conflict of interest statement

The authors declare that the research was conducted in the absence of any commercial or financial relationships that could be construed as a potential conflict of interest.
