# Supplementary material for: LRP1 Modulates APP Intraneuronal Transport and Processing in Its Monomeric and Dimeric State
Source: Front Mol Neurosci. 2017 Apr 27;10:118. doi: 10.3389/fnmol.2017.00118 (PMC5406469; doi:10.3389/fnmol.2017.00118)
Supplement: Supplementary file 3 [file DataSheet1.PDF]

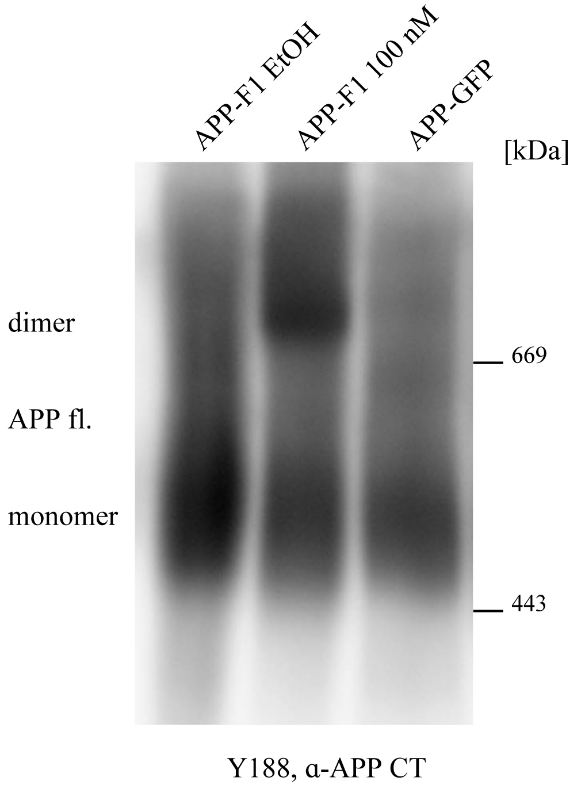

Supplementary Figure 1: Blue native gel analysis of APP-GFP in comparison to APP-F1. APP-GFP or APP-F1 (containing one FKBP domain fused to the C-terminus of APP) were heterologously expressed in N2a cells. 18-20 hours after transfection and 1 hour prior harvesting, APP-F1 expressing cells were treated with 100 nM AP20187 (dimerizer), which binds two FKBP molecules to induce dimerization of APP as described in Eggert et al. (2009). Treatment with the vehicle of the dimerizer, ethanol, served as a negative control. Membrane fractions of indicated cells were analyzed via blue native gel analysis (Eggert et al., 2009). Antibody Y188 (Abcam) directed against the C-terminus of APP was used to detect monomeric and dimeric APP. Note the predominantly monomeric state of APP-GFP and ethanol treated APP-F1 cells in comparison to APP-F1 cells treated with dimerizer.

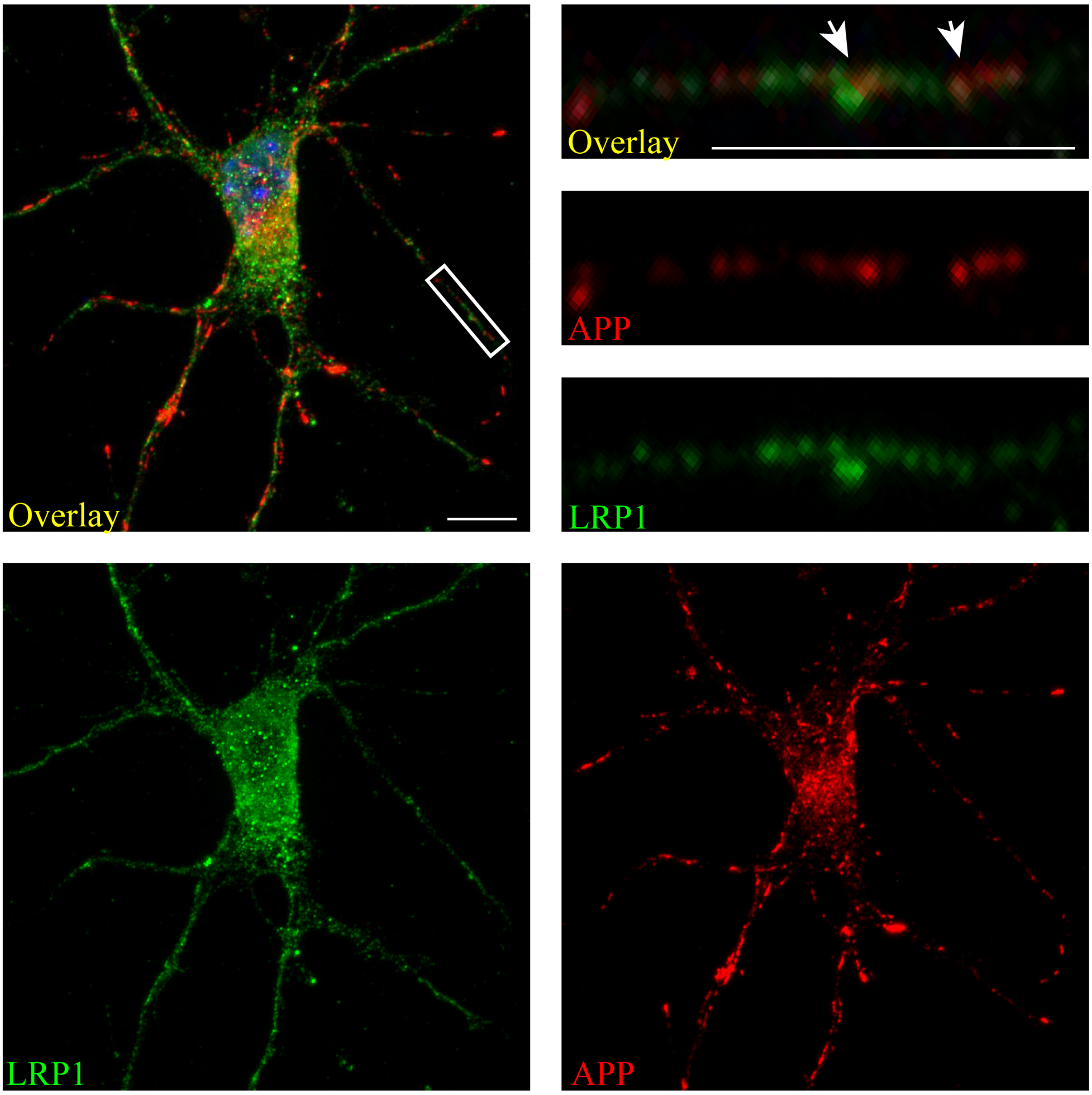

Supplementary Figure 2: Co-localization of LRP1 and APP in primary cortical neurons. Immunocytochemical analysis of primary cortical neurons differentiated for 7 days in vitro. Endogenous APP (red) and LRP1 (green) were stained with C1/6.1 and 1704 [Pietrzik et al., 2002], respectively. Hoechst 33258 was used for nuclear counterstaining (blue). Co-localization is indicated in yellow. The outlined region in the overlay is enlarged in the upper right panels. Please note the high degree of co-localization of APP and LRP1 immunoreactivity in the cytoplasmic region and the low partial overlap in neurites (indicated by arrow heads). Scale bar: 5 µm.

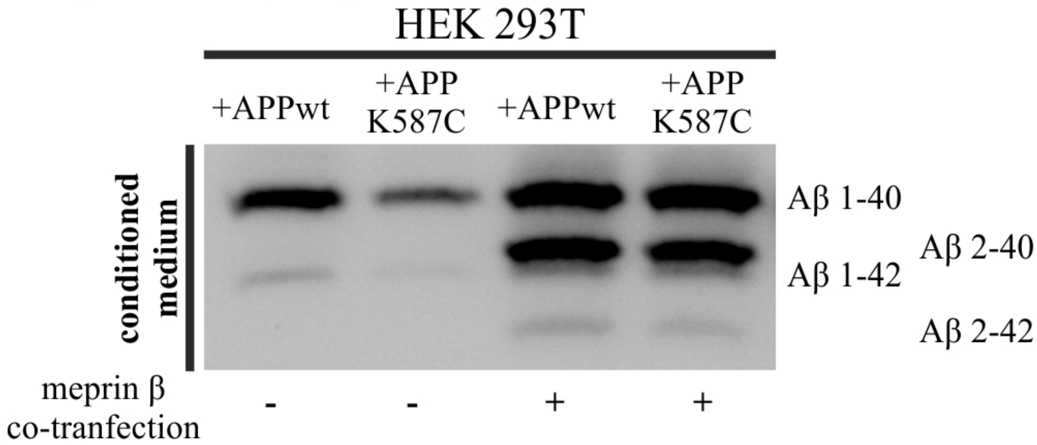

Supplementary Figure 3: Meprin β overexpression affects Aβ generation. Urea SDS-PAGE of immunoprecipitated Aβ from the conditioned medium of HEK 293T cells 24 hours after transfection. Peptides were detected by the IC16 antibody (1:500). Cells were transiently transfected with either APP695 wt or APP695 K587C or co-transfected with meprin β. Transfections with the APP constructs alone served as controls. In APP695 K587C transfected cells additionally expressing meprin β, levels of truncated Aβ species (2-40 and 2-42) were raised to the same amount as deserved for cells transfected with APP695 wt and meprin β. Note, that in the corresponding controls Aβ generation from the APP dimer bearing construct was reduced compared to that derived from wildtype APP.
